# Giving to others and neural processing during adolescence

**DOI:** 10.1016/j.dcn.2022.101128

**Published:** 2022-06-22

**Authors:** Maira Karan, Lee Lazar, Carrianne J. Leschak, Adriana Galván, Naomi I. Eisenberger, Jessica P. Uy, Macrina C. Dieffenbach, Eveline A. Crone, Eva H. Telzer, Andrew J. Fuligni

**Affiliations:** a Department of Psychology, UCLA, Los Angeles, CA 90095, USA; b Erasmus University Rotterdam, Rotterdam, The Netherlands; c Department of Psychology, University of North Carolina, Chapel Hill, NC 27599, USA; d Jane and Terry Semel Institute for Neuroscience and Human Behavior, UCLA, 760 Westwood Plaza, Los Angeles, CA 90095, USA

**Keywords:** Adolescence, Prosocial behavior, Brain development, FMRI, Decision-making

## Abstract

Adolescence is marked by an increased sensitivity to the social environment as youth navigate evolving relationships with family, friends, and communities. Prosocial behavior becomes more differentiated such that older adolescents increasingly give more to known others (e.g., family, friends) than to strangers. This differentiation may be linked with changes in neural processing among brain regions implicated in social decision-making. A total of 269 adolescents from 9–15 and 19–20 years of age completed a decision-making task in which they could give money to caregivers, friends, and strangers while undergoing functional magnetic resonance imaging (fMRI). Giving to caregivers and friends (at a cost to oneself) increased with age, but giving to strangers remained lower and stable across age. Brain regions implicated in cognitive control (dorsolateral and ventrolateral prefrontal cortex) showed increased blood-oxygen-level-dependent (BOLD) activation with increasing age across giving decisions to all recipients; regions associated with reward processing (ventral striatum and ventral tegmental area) showed increased activation across all ages when giving to all recipients. Brain regions associated with social cognition were either not active (dorsomedial prefrontal cortex) or showed reduced activation (temporal parietal junction and posterior superior temporal sulcus) when giving to others across all ages. Findings have implications for understanding the role of brain development in the increased complexity of social decision-making during adolescence.

Adolescence is marked by an increased sensitivity to the social environment as youth navigate evolving relationships with their family, friends, and communities while undergoing critical behavioral and neural maturation. Prosocial behavior—generally defined as voluntary acts with the intention of benefiting others—is an important component of creating and maintaining social relationships with others that has been linked with better psychological and physical health (Fuligni, 2019, Padilla-Walker and Carlo, 2014). Studies have highlighted how prosocial behavior becomes more differentiated with age, increasingly depending upon factors such as the intended recipient of the actions (Güroğlu et al., 2014; Padilla‐Walker et al., 2018). It is possible that this differentiation may be linked with key changes in the adolescent brain that have been tied to more sophisticated decision-making, particularly in the social realm (Blakemore and Mills, 2014, Crone and Dahl, 2012, Crone and Fuligni, 2020). The current study examined age differences in adolescents’ giving behavior toward caregivers, friends, and strangers and investigated how neural regions implicated in cognitive control, social cognition, and reward processing tracked with differences in giving across age and recipient.

Compared to younger children, adolescents’ prosocial behavior becomes more complex and dependent upon situational factors as adolescents’ social reasoning becomes more flexible (Carlo, 2013, Crone and Dahl, 2012). One source of increased differentiation is the target or recipient of the prosocial act. For example, Güroğlu et al. (2014) observed that whereas 9 year-old children gave resources at a cost to themselves equally to close friends and strangers, older adolescents (15 and 18 years) increasingly gave more to friends than to strangers. Padilla-Walker et al. (2018) obtained similar patterns in a longitudinal study of adolescents’ self-reports of giving support and helping others, and additionally found that prosocial behaviors directed towards family remained stable and then increased in late adolescence. Distinctions in adolescent prosocial behavior by target has been observed in other studies and has been argued to be due to adolescents’ increasingly complex social reasoning such as a preference for known others and understanding the role of reciprocity in close relationships (Fehr et al., 2013, Güroğlu et al., 2014, van de Groep et al., 2020b).

Decisions to provide support and resources to others can involve critical social reasoning and the consideration of multiple factors such as the cost to oneself, the mental states and needs of others, and the potential feelings of satisfaction and reward experienced from helping others (Keltner et al., 2014). As such, the decision-making by which individuals give to others has been tied to several neural regions in networks associated with cognitive control, social cognition, and reward processing that undergo significant change during adolescence (Bellucci et al., 2020a, Crone and Fuligni, 2020, Eisenberger, 2013a, Keltner et al., 2014). Giving resources to family or strangers has been linked with neural activation in region associated with social cognition such as the dorsomedial prefrontal cortex (dmPFC) and posterior superior temporal sulcus (pSTS) as well as regions associated with cognitive control, such as dorsolateral prefrontal cortex (dlPFC), and ventrolateral prefrontal cortex (vlPFC). These same prosocial behaviors involving friends have been associated with activation in regions associated with social cognition, such as the temporal parietal junction (TPJ), pSTS, and medial prefrontal cortex (mPFC) as well as reward processing regions, such as ventral striatum (VS), and nucleus accumbens (NAcc) (Braams et al., 2014a, Braams and Crone, 2017a, Braams and Crone, 2017b, Brandner et al., 2020, Brandner et al., 2021, Do et al., 2019, Schreuders et al., 2021, Spaans et al., 2018, Spaans et al., 2019, Telzer et al., 2011, Telzer et al., 2013, van Hoorn et al., 2016). Additionally, giving money to one’s family at a loss to oneself has been associated with increased activation in reward-related neural regions, such as the VS and ventral tegmental area (VTA) at levels equal to or greater than when the adolescents receive money for themselves (Telzer et al., 2010, Telzer et al., 2011, Telzer et al., 2013).

Although these studies highlight the importance of networks associated with cognitive control, social cognition, and reward processing when giving to others, previous research generally has assessed giving to only a single recipient. Little is known about the role of these neural networks in the increased preference in giving to known others such as family and friends as compared to strangers during adolescence. Given the general pattern of greater activation in those regions when giving, one might predict that increased giving to family and friends versus strangers across adolescence would be associated with greater neural activation when giving to family and friends. Consistent with this expectation, studies of middle and late adolescents/young adults have reported greater activation in regions associated with cognitive control, social cognition, and reward processing when giving to friends compared to strangers (Schreuders et al., 2019, van de Groep et al., 2020a). Yet these studies did not examine age differences and it is unclear whether the differential activation according to the recipient becomes greater across adolescence along with the increased differentiation in giving. A recent study that examined a wider age span (9–19 years of age) observed increased preference for giving to friends versus strangers across adolescence but did not find age differences in the differentiation of neural activation when giving to each recipient (van de Groep et al., 2022). Instead, the most notable age difference was among older adolescents who demonstrated increased engagement of brain regions associated with cognitive control in the prefrontal cortex (PFC) when they made giving decisions, regardless of the recipient. The pattern of increased engagement of the PFC with age is consistent with models that emphasize the enhanced role of the PFC in many aspects of decision-making during adolescence, including the flexibility and need to balance self and others that are central to the complexity of prosocial behavior throughout the adolescent period (Crone and Dahl, 2012, Crone and Fuligni, 2020).

## 1 Current study

The current study examined the prosocial behavior and associated neural processing of children, adolescents, and young adults while they completed a prosocial decision-making task for three different target recipients (caregiver, friend, and stranger) while undergoing functional magnetic resonance imaging (fMRI). This investigation was guided by two primary aims: (1) to assess the effects of age and giving target on prosocial behavior, and (2) to identify the neural correlates of prosocial behavior as a function of age and giving target. In terms of behavior, we expected that giving would increase with age toward friends, increase or remain stable across age toward caregivers, and decrease or remain stable with age toward strangers. With respect to the brain, we hypothesized two possible outcomes. First, as suggested by recent studies (Schreuders et al., 2019, van de Groep et al., 2020a), we predicted increased differentiation of activation according to target, with greater activation in regions in the cognitive control (dlPFC, vlPFC), social cognition (dmPFC, TPJ, pSTS), and reward processing (VS, VTA) networks when giving to caregivers and friends as compared to strangers. Alternatively, as was found by van de Groep et al. (2022) in the PFC, there could be increased activation irrespective of target in regions associated with cognitive control, reward processing, and social cognition when giving to others across age given the significant developments and enhanced role of these three networks in decision-making in general during the adolescent period.

## 2 Method

### 2.1 Participants

Adolescents between the ages of 9–15 years were recruited via flyers, advertisements, and through class presentations to schools within the Los Angeles Unified School District. Participants were also recruited from the Clinical and Translational Science Institute (CTSI) database of families in the University of California Los Angeles (UCLA) and affiliated medical systems. Additionally, participants aged 19–20 years were recruited from undergraduate classes at UCLA in order to include older adolescents in the estimates of age differences in behavior and neural activation. All participants were right-handed, fluent in English, free of MRI contraindications, had no previous psychiatric diagnoses, and were not pregnant or trying to become pregnant at the time of the study session. Participants came from one of two parent studies: approximately half of participants (*N* = 134) participated in a cross-sectional study (Study 1), and 140 participants completed the present measures as part of the first wave of a longitudinal study (Study 2). With the exception of slightly different scanning parameters for the structural MRI image (detailed below), the study protocol and task procedures for these two studies were identical.

A total of 274 participants were enrolled to participate in the current investigation (Study 1 + Study 2), but 5 participants’ data were excluded from all analyses because they either (1) misunderstood the task (n = 4) or (2) the button box malfunctioned (n = 1). Thus, 269 participants were included in the behavioral analysis, which consisted of adolescents aged 9 (n = 43), 10 (n = 37), 11 (n = 32), 12 (n = 33), 13 (n = 33), 14 (n = 32), 15 (n = 20), 19 (n = 21) and 20 (n = 18) years old. Of the 269 participants with behavioral data, 43 participants were excluded from neuroimaging analyses because (1) they did not complete a scanning session (n = 3), (2) the projector malfunctioned and data was collected behaviorally only (n = 1), (3) a brain abnormality was detected from the participant’s scan (n = 1), (4) the participant had braces and data was collected behaviorally (n = 1), or (5) due to excessive motion (n = 7) or due to poor image quality (n = 30), leaving a final neuroimaging sample of 226 participants. Sample sizes varied by region of interest (ROI) analyses due to drop out as well (see Supplemental Information).

Participants were approximately half female (46.5% female in the behavioral sample; 47.5% in neuroimaging sample). The self-reported ethnic composition of the behavioral sample was 31.8% European American, 21.3% Multi-ethnic, 19.4% Hispanic/Latinx, 11.2% Asian American, 8.1% African American, 7.4% Other, and 0.7% Native American. Averaging across both caregivers’ level of education indicated that caregivers averaged between “some college” and “graduated from college”. Parents reported a wide range of household incomes ($15k - $3M) with 16% reporting up to $50k per year, 21% between $50k and $100k, 29% between $100k and $200k, and 33% over $200k. Demographics for the neuroimaging sample were similar to the behavioral sample. Parents and youth provided written consent and assent in accordance with UCLA’s Office of the Human Research Protection Program (OHRPP).

### 2.2 Giving task paradigm

While undergoing fMRI scans, participants completed a costly giving task that has been used in previous research to assess prosocial decision-making (Telzer et al., 2013, Telzer et al., 2014, Telzer et al., 2015). Prior to learning about the task, participants were asked to select a friend and a caregiver, without being told that they would later earn money for them. The precise prompts are available in the Supplemental Information.

**Fig. 1:**
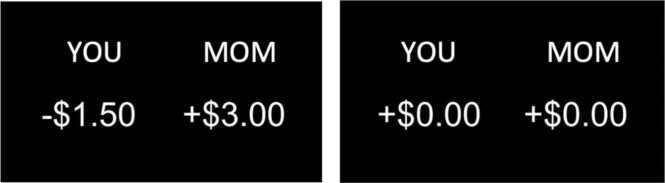
An example of a Costly Giving (left) and Control trial (right). The relevant target of the game (caregiver, friend, or stranger) was indicated at the top right.

In this task, participants responded to a series of financial offers in which they could earn money for themselves, their chosen caregiver and friend as well as a future participant in the study who was unknown to the participant (stranger). Participants played three rounds of the task, one for each target recipient (caregiver, friend, or stranger). Four types of offers were presented during task: (1) Costly Giving (40 trials per target), in which the recipient earned money at a cost to the participant (e.g., YOU -$1.00, OTHER +$3.00), (2) Non-Costly Rewards (16 trials per target), in which participants earned money without a cost to the other person (e.g., YOU +$3.00, OTHER -$0.00), (3) Non-Costly Giving (5 trials per target), in which the other person earned money without a cost to the participant (e.g., YOU -$0.00, OTHER +$3.00), (4) and Control trials (16 trials per target), in which neither the participant nor the other person gained or lost any money (e.g., YOU -$0.00, OTHER -$0.00). Participants were told to accept or reject these offers using a handheld button box. Additionally, they were informed that a few trials would be randomly selected at the end of the task that would determine how much each recipient and the participant had earned. Costly Giving trials accepted by the participants were operationalized as giving behavior and is the primary trial type of interest in the present study. We compared Costly Giving trials to Control trials, which controlled for the visual and motor aspects of the task (Fig. 1). Costly Giving trials were compared to Control trials that were presented within the same run of the task. The additional trial types were included to provide variation in decision-making trials and keep participants engaged and interested throughout the task.

Offer values ranged in increments of $0.25 from –$3.75 to $0 and + $2.00 to + $7.00 for the participant, and $0, + $2.00 to + $7.00 for the recipient, to reduce heuristic responding and fatigue from the task (Andreoni and Miller, 2002, Harbaugh et al., 2007). Costly Giving trials varied in terms of the gain to loss ratio in order to vary the difficulty of the decisions and obtain a wider range of individual differences in responses; the gain was always greater than the loss. Each offer was presented for 3 s, during which participants could accept or reject the offer, followed by a jittered fixation (500–4000 ms, ms). At the end of the task, 10 randomly chosen trials were selected and participants were paid their earnings in cash based on the outcome of these trials. Participants were given three separate payments—earnings won for themselves, their friend, and their caregiver. Earnings won for the stranger were given to the next participant in the study as a part of their compensation for completing the study.

### 2.3 fMRI data acquisition

Imaging data were acquired on a Siemens Prisma 3-Tesla MRI scanner housed at UCLA’s Staglin International Mental Health Research Organization Center for Cognitive Neuroscience. Foam padding was placed around each participant’s head for comfort and to constrain head movement. The task was presented via a projector that participants viewed through a mirror attached to the head coil.

For each participant, an initial set of three (one in each plane: coronal, sagittal, axial) 2D structural scout (localizer) gradient-echo images (TR=3.15 ms, TE=1.37 ms, matrix size=160 × 160, FoV=260 mm, 128 slices, flip angle=8°, 1.6-mm thick, 1.6-mm inplane resolution, 0.32-mm gap) was acquired in order to enable prescription of slices obtained in structural and functional scans. A T1-weighted magnetization prepared rapid gradient echo (MPRAGE) structural scan (parameters for participants from Study 1: TR=1900 ms, TE=2.26 ms, matrix size=256 × 256, FoV=250 mm, 176 slices, flip angle=9°, 1-mm thick, 1-mm inplane resolution, 0.5-mm gap; parameters for participants from Study 2: TR=2000 ms, TE=2.52 ms, matrix size=256 × 256, FoV=256 mm, 192 slices, flip angle=12°, 1-mm thick, 1-mm inplane resolution, 0.5-mm gap), coplanar with the functional scans, was collected for all participants.

The giving task consisted of three functional (echo planar T2 * -weighted gradient-echo) MRI scans. Each functional run (TR=2000 ms, TE=30 ms, matrix size=64 × 64, FoV=192 mm, 34 slices, flip angle=90°, 4-mm thick, 3-mm inplane resolution, no gap) lasted 6 min and 40 s

### 2.4 fMRI data preprocessing and analysis

*fMRI data preprocessing*. fMRI data was preprocessed using Statistical Parametric Mapping 12 (SPM12; Welcome Department of Cognitive Neurology, Institute of Neurology, London, England). For each subject, functional images were realigned to the mean functional image and resliced to correct for head motion. Afterward, the subject’s MPRAGE was segmented and bias-corrected. Deformation fields were computed for normalizing the MPRAGE to Montreal Neurological Institute (MNI) space. Functional images were co-registered to the bias-corrected structural grey matter. All images were then affine registered into Montreal Neurological Institute space. The previously generated deformation fields were used to normalize all images into MNI space, with functional images undergoing integrated spatial smoothing (5 mm, Gaussian kernel, full width at half maximum).

*fMRI Data Analysis.* Following pre-processing, a general linear model (GLM) was constructed for each participant in which the task was modeled as an event-related design. The time series was high-pass filtered using a 128 Hz function, and serial autocorrelation was modeled as an AR(1) process. In cases where motion of more than 1 mm frame-wise displacement was detected, individual nuisance regressors were added to remove such images from analyses. Each individual run (for caregiver, friend, and stranger) was entered into the model as separate sessions. Each active condition (Costly Giving, Non-Costly Giving, Non-Costly Reward, Control) within a run was modeled in separate regressors. Given that trials in which offers were accepted were of primary interest in the present study, accepted trials for each condition were modeled in separate regressors so they could be separately examined. Control trials were modeled in a single regressor, regardless of whether the trial was accepted or rejected, given that the financial outcome in these trials was identical. A linear contrast comparing accepted Costly Giving trials to Control trials within the same run was computed for each participant in order to examine prosocial giving behavior.

*Definition of Regions of Interest.* A set of a priori regions of interest (ROIs) associated with cognitive control, social cognition, and reward processing were selected for analysis. ROIs associated with cognitive control included the bilateral dlPFC and vlPFC. The dlPFC ROI was anatomically defined by the Wake Forest University (WFU) PickAtlas (Maldjian et al., 2003), and the bilateral vlPFC was anatomically defined by the Harvard-Oxford Cortical Structural Atlas probability map implemented in the fMRIB Software Library (FSL) that was then thresholded at 25% probability. ROIs associated with social cognition included the bilateral dmPFC, TPJ, and pSTS. The dmPFC ROI was defined using Neurosynth by searching and downloading the dmPFC region in the automated meta-analysis tool and then masking this with the medial frontal gyrus from the WFU PickAtlas, based on prior work (Maldjian et al., 2003, Yarkoni et al., 2011). The TPJ ROI was created by combining the right TPJ, comprised of 2812 voxels all z > 6 mm, centered at [54 − 52 23] and the left TPJ, comprised of 2444 voxels all z > 6 mm centered at [− 52 − 58 25], following past work (Dufour et al., 2013). The pSTS ROI was created by extending the Desikan-Killiany Atlas (Desikan et al., 2006) defined bank superior temporal sulcus to the border of the TPJ (Mills et al., 2014). Finally, ROIs associated with reward processing included the bilateral VS and VTA. The VS ROI was defined by combining the caudate and putamen from the AAL atlas and constraining the ROI at − 24 < *x* < 24, 4 < *y* < 18, and − 12 < *z* < 0, following past work (Inagaki and Eisenberger, 2012). The VTA ROI was defined based on localizations from prior work utilizing an 8 mm sphere centered on [0 − 18 − 18] (de Greck et al., 2008). All ROIs used in this study can be viewed and downloaded in Neurovault (https://identifiers.org/neurovault.collection:12218). The dlPFC, TPJ, dmPFC and TPJ ROIs were based on work from Telzer and colleagues that can be found on Neurovault as well (https://neurovault.org/collections/SISNGRAB/).

Mean parameter estimates were extracted from the ROIs for each participant and entered into standard statistical software (see below) for further analysis. Whole-brain analyses were conducted using a voxel-wise height threshold of *p* < .001 (uncorrected) combined with a cluster-level extent threshold of *p* < 0.05, corrected for multiple comparisons using the family-wise error (FWE) rate.

### 2.5 Analysis plan

Costly giving behavior was computed as the number of trials that participants decided to accept divided by the number of trials participants responded to (responses of accept and reject only). Trials that the participant did not respond to were excluded from the denominator. Giving behavior was analyzed in two-level multilevel models such that target runs (caregiver, friend, stranger) were nested within individuals. Initial models examined main effects of target (caregiver and friend vs. stranger baseline) and linear age (mean centered at 13.08 years) in the same model, and quadratic age was added in a subsequent model to assess possible nonlinear age associations. Follow-up models separately examined target × linear age and target × quadratic age interactions.

Behavioral reaction time data (measured in seconds) for costly giving and control trials were analyzed in three-level multilevel models such that trials were nested within targets that were nested within individuals. Initial models examined main effects of response type (accept = 0, reject = 1), linear age, and target (caregiver and friend vs. stranger baseline). Follow-up models examined quadratic age, two-way interactions between target and age (mean centered linear and quadratic), as well as three-way interactions between target, age (mean centered linear and quadratic), and response type.

ROI parameters of mean activation were extracted and analyzed in two-level models such that target runs were nested within individuals. In order to avoid unreliable estimates of neural signal during costly giving, the target-specific data for participants who accepted fewer than 7 Costly Giving trials for that target were excluded. Multilevel modeling accounted for these missing data and allowed us to retain those participants’ data for other targets if they had at least 7 accepted trials for those targets. Therefore, sample sizes for the ROI analyses varied and are listed in the Supplemental Information. As with the behavioral data, initial models examined main effects of target (caregiver and friend vs. stranger baseline) and linear age (mean centered) in the same model, and quadratic age was added in a subsequent model to assess potential nonlinear associations with age. Separate follow-up models examined target by linear age and target by quadratic age interactions. A Bonferroni correction was used to account for the analysis of seven separate ROIs. With a family-wise error rate of *p* < .05, effects from these models had to be *p* < .007 in order to achieve statistical significance. Uncorrected results are provided in the Supplemental Information, but they are not discussed here. Analyses were run on STATA 15.1 (College Station, TX).

Whole-brain parameter values were examined by entering linear age, quadratic age, and target as regressors in a GLM. Analyses were run on SPM12.

All models were re-run to control for sex, ethnicity, and parent education. However, none of these covariates were found to be associated with the outcome variables of interest. Thus, results are reported from models that exclude these covariates.

## 3 Results

### 3.1 Costly giving behavior

**Fig. 2:**
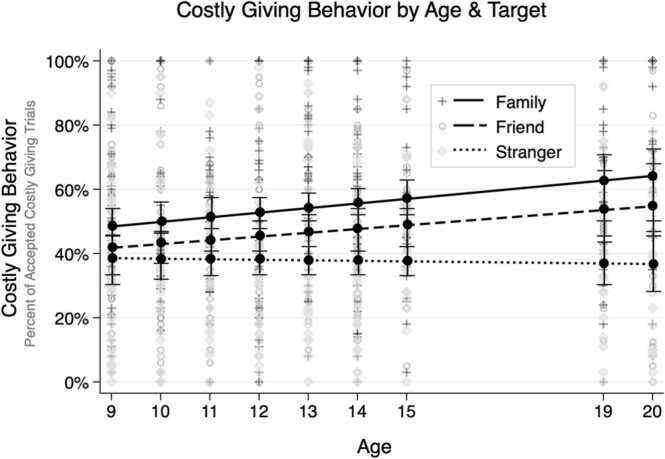
Costly Giving Behavior by Linear Age and Target Giving behavior, calculated as the percentage of costly giving trials accepted, according to age and target. Bars reflect the standard error at each age. The sample did not include adolescents who were ages 16–18 years old. Data points are observed costly giving according to target (see legend).

As shown in Fig. 2, age differences in giving behavior varied according to the target recipient. Significant target × age interactions (caregiver × age: *b* = 0.01, *SE* =0.004, *p* = .001; friend × age: *b* = 0.01, *SE* =0.004, *p* = .030) were obtained, along with a significant intercept (*b* = 0.37, *SE* = 0.02, *p* < .001) and main effects of target (family: *b* = 0.17, *SE* = 0.01, *p* < .001; friend: *b* = 0.09, *SE* = 0.01, *p* < .001). Follow-up simple slope analyses of age effects for each target indicated that giving to caregivers (*b* = 0.01, *SE* = 0.01, *p* = .018) and friends (*b* = 0.01, *SE* = 0.01, *p* = .012) was positively associated with age whereas there were no age differences when giving to strangers (*b* = –0.001, *SE* = 0.01, *p* = .889). Contrasts of target at each age indicated that there was no significant difference in giving behavior by target among 9 year-olds, but by 10 years and up youth gave significantly more to caregivers than to strangers, and by 12 years and up youth gave significantly more to friends than to strangers (*p*s = 0.01). Other models suggested no significant non-linear associations with age nor any interactions of non-linear age with target. All findings were maintained when restricting analyses to the neuroimaging subsample.

### 3.2 Costly giving behavior: reaction times

A main effect of linear age (*b* = –0.01, *SE* = 0.00, *p* = .002) and target (caregiver: *b* = –0.04, *SE* = 0.02, *p* = .023) emerged when examining reaction times of giving (accepting costly giving trials) during the task indicating that older youth were faster to give to others irrespective of target, and irrespective of age youth were faster to give to caregivers than to strangers. A main effect of linear age (*b* = –0.02, *SE* = 0.00, *p* < .001) and target (friend: *b* = 0.08, *SE* = 0.02, *p* = .001) emerged when examining reaction times for accepting control trials during the task. Faster reaction times were associated with older adolescent age when youth chose to accept control trials, irrespective of target. Slower reaction times emerged when youth, irrespective of age, accepted control trials for friends compared to strangers. All results were maintained when restricting analyses to the neuroimaging subsample.

## 4 Neuroimaging results

### 4.1 Region of interest analyses

**Fig. 3:**
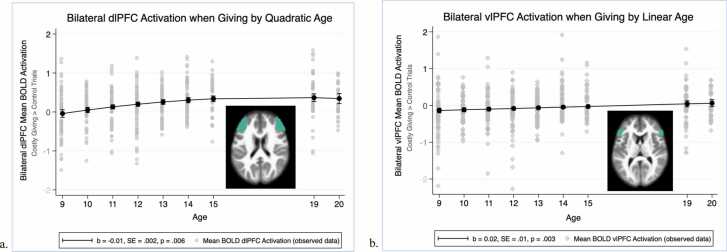
Neural activation in brain regions associated with cognitive control. a. Quadratic age trend of bilateral dorsolateral prefrontal cortex (dlPFC) BOLD activation comparing Costly Giving trials > Control trials. b. Linear age trend of bilateral ventrolateral prefrontal cortex (vlPFC) BOLD activation comparing Costly Giving trials > Control trials.

*Regions associated with cognitive control.* Activation in the bilateral dlPFC and vlPFC during costly giving, compared to control, increased with age and with no variation according to the target recipient (see Fig. 3a and b). First, a main effect of quadratic age (linear age: *b* = 0.05, *SE* = 0.01, *p* < .001; quadratic age: *b* = –0.01, *SE* = 0.002, *p* = .006) and a significant intercept (*b* = 0.31, *SE* = 0.04, *p* < .001) emerged in the model examining bilateral dlPFC activation, but there were no main effects of target (caregiver: *b* = –0.09, *SE* = 0.05, *p* = .064; friend: *b* = –0.07, *SE* = 0.05, *p* = .191). As shown in Fig. 3a, bilateral dlPFC activation across all targets increased until about 15 years and remained stable between ages 15–20 years while giving. Second, a main effect of linear age (linear age: *b* = 0.02, *SE* = 0.01, *p* = .003) emerged in the model examining bilateral vlPFC activation, but again there were no main effects of target (caregiver: *b* = –0.05, *SE* = 0.05, *p* = .336; friend: *b* = −0.06, *SE* = 0.05, *p* = .234). As shown in Fig. 3b, bilateral vlPFC activation across all targets increased steadily until age 20 years. Follow-up models suggested no quadratic effect of age for bilateral vlPFC nor any interactions between target and linear or quadratic age for either the dlPFC or vlPFC.

**Fig. 4:**
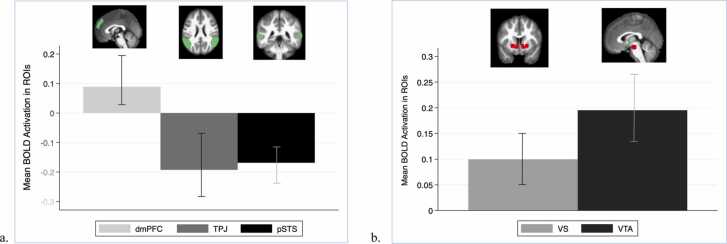
Mean BOLD Activation in Social Cognition and Reward-Related ROIs. a. Mean BOLD activation in the dorsomedial prefrontal cortex (dmPFC), bilateral temporal parietal junction (TPJ), and bilateral posterior superior temporal sulcus (pSTS), regions associated with social cognition, comparing Costly Giving trials > Control trials. b. Mean BOLD activation in the bilateral ventral striatum (VS) and ventral tegmental area, regions associated with reward processing, during Costly Giving trials > Control trials.

*Regions associated with social cognition.* Activation in the bilateral TPJ and pSTS demonstrated lower activation during costly giving to others as compared to control, with no differences across age and no variation according to the target recipient (Fig. 4A). There was a significant intercept (*b* = –0.19, *SE* = 0.04, *p* < .001) in the bilateral TPJ model, indicating lower activation while giving, but no main effects of age (linear age: *b* = –0.01, *SE* = 0.01, *p* = .107) or target (caregiver: *b* = –0.01, *SE* = 0.05, *p* = .926; friend: *b* = –0.01, *SE* = 0.05, *p* = .880). Similarly, there was a significant intercept (*b* = –0.17, *SE* = 0.04, *p* < .001) in the pSTS model indicating lower activation while giving, but no main effects of age (linear age: *b* = –0.01, *SE* = 0.01, *p* = .238) or target (caregiver: *b* = –0.03, *SE* = 0.05, *p* = .589; friend: *b* = –0.05, *SE* = 0.05, *p* = .350). In contrast, results indicated no differential activation in the dmPFC during costly giving to others as compared to control (intercept: *b* = 0.09, *SE* = 0.04, *p* = .025; non-significant after Bonferroni correction), and no variation according to linear age (*b* = 0.01, *SE* = 0.01, *p* = .447) or target (caregiver: *b* = –0.11, *SE* = 0.05, *p* = .035; friend: *b* = –0.08, *SE* = 0.05, *p* = .116). Follow-up models suggested no quadratic effects of age nor any interactions between target and linear or quadratic age for any of the ROIs associated with social cognition.

*Regions associated with reward processing.* Activation in the bilateral VS and VTA demonstrated more activation during costly giving to others as compared to control, with no differences across age and no variation according to the target recipient (Fig. 4B). There was a significant intercept (*b* = 0.13, *SE* = 0.03, *p* < .001) in the bilateral VS model, indicating more activation while giving, but no main effects of age (linear age: *b* = 0.01, *SE* = 0.004, *p* = .015) or target (caregiver: *b* = –0.05, *SE* = 0.03, *p* = .108; friend: *b* = –0.02, *SE* = 0.03, *p* = .607). Similarly, there was a significant intercept (*b* = 0.18, *SE* = 0.03, *p* < .001) in the VTA model, indicating more activation while giving, but no main effects of age (linear age: *b* = 0.01, *SE* = 0.005, *p* = .255) or target (caregiver: *b* = –0.04, *SE* = 0.04, *p* = .321; friend: *b* = –0.03, *SE* = 0.04, *p* = .410). Follow-up models suggested no quadratic effects of age nor any interactions between target and linear or quadratic age for the bilateral VS and VTA.

### 4.2 Whole brain analyses

**Fig. 5:**
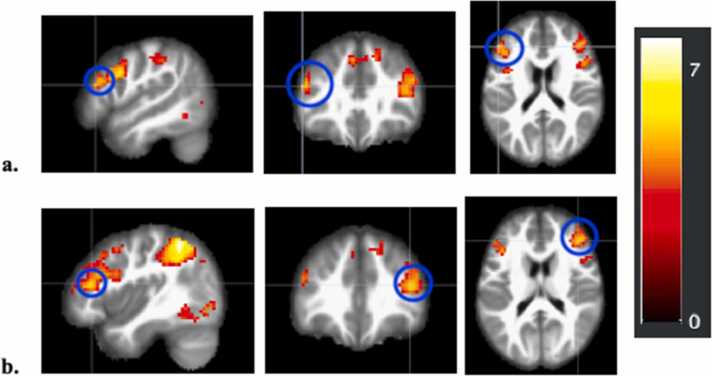
*Whole Brain Heat Maps of Bilateral dlPFC BOLD Activation during Giving* a. Whole brain activation map highlighting (blue circles) the left dlPFC positively associated with linear age for Costly Giving trials > Control trials collapsed across giving target (*t*(212) = 4.92, *p* < .001, MNI Coordinates: –51, 32, 20). b. Whole brain activation map highlighting (blue circles) the right dlPFC positively associated with linear age for Costly Giving trials > Control trials collapsed across giving target (*t*(212) = 5.31, *p* < .001, MNI Coordinates: 42, 35, 17).

Results revealed activation in several brain regions at the whole brain group level that were positively associated with linear age for Costly Giving Trials > Control Trials (see Supplemental Information, Table S4). In line with our hypotheses, activation in bilateral dlPFC increased with adolescent age when giving (collapsed across giving target) (right dlPFC: *t*(212) = 5.31, *p* < .001, MNI Coordinates: 42, 35, 17; left dlPFC: *t*(212) = 4.92, *p* < .001, MNI Coordinates: –48, 8, 29) (Fig. 5). Whole brain clusters did not survive when examining contrasts comparing target conditions. See Supplemental Information for a full table of results.

## 5 Discussion

Advances in social reasoning and decision-making across the adolescent years are accompanied by increased complexity in prosocial behavior toward others. Our findings suggest that one type of complexity is greater differentiation in costly giving to others, with greater preference for known others such as caregivers and friends over strangers with age. Neural activation when giving did not differ according to the target, but two regions associated with cognitive control and decision-making – the dlPFC and vlPFC – became progressively more engaged across age as adolescents increasingly differentiated their giving behavior according to target. Regions associated with reward processing were equally active when giving to all targets and across all ages, but those linked with social cognition were either not active or less engaged when giving to others. Results highlight the role played by key networks of the developing brain in the emergent sophistication and flexibility in social reasoning during the adolescent years.

As hypothesized, results show increased differentiation in costly giving according to the recipient across adolescence. At nine years of age, participants gave money (at a loss to themselves) to caregivers, friends, and strangers at an equal rate. After that point, adolescents increasingly gave more to caregivers and friends such that by older ages, they preferred to give more to caregivers than friends, and more to both of these recipients than to strangers. By examining both the behavior and neural responses associated with giving to caregivers, friends, and strangers during early and late adolescence, this investigation extended previous studies’ aims that have compared fewer giving targets or excluded neuroimaging. Adolescents’ greater preference for providing resources and support to both family and friends with increasing age, likely reflecting a move away from basic allocation rules such as equality and an enhanced understanding of the role of mutuality and reciprocity within close relationships (Fehr et al., 2013, van de Groep et al., 2020b, Güroğlu et al., 2014; Padilla‐Walker et al., 2018). At the same time, the developmental patterns suggest increased parochialism and potentially in-group favoritism in prosociality that may be less desirable from a broader, societal perspective (Fehr et al., 2013).

The significant patterns of activation in brain regions associated with cognitive control, social cognition, and reward processing highlight the important role played by developments in these networks for prosocial behavior during adolescence. Neural activation when giving to others increased with age in regions associated with cognitive control. The dlPFC showed no activation at 9 years of age but became increasingly active until 15 years, whereas activation in the vlPFC increased with age from below to above zero at 19 and 20 years of age. Age-related increases in the dlPFC were also demonstrated in the whole-brain analyses. Similar results were also reported in a recent study by (van de Groep et al., 2022) and highlight the increased importance of regions in the lateral PFC for prosocial decision-making through the adolescent years. These regions have been implicated in the planning and inhibition of self-maximizing impulses when engaging in costly prosocial behavior (Bellucci et al., 2020b, Crone and Fuligni, 2020). Given that the current prosocial task involved sacrificing one’s own interest for the benefit of another with a costly donation, it is possible adolescents increasingly engaged in these regulatory processes as they became more likely with age to discriminate between giving to known others versus strangers. This discrimination is one example of the more complex social decision-making during adolescence that the lateral PFC has been suggested to subserve (Crone and Dahl, 2012), and the increases in activation of cognitive control regions with age may reflect the relatively slow maturation and increased use of neural regions associated with cognitive control in social reasoning (Dumontheil et al., 2012). The current findings underscore the importance of the PFC in the development of prosocial behavior during adolescence.

The significant activation in both the VS and VTA dovetails with several previous studies highlighting the engagement of regions associated with reward processing when giving resources to others, particularly at a cost to oneself (Telzer et al., 2010, Telzer et al., 2011, Telzer et al., 2013). Activation in these regions may be due to the motivation to provide support and resources to others, as evidenced by the tendency for both adolescents and adults to give at least some resources to others in resource allocation tasks such as that used in the present study (Engel, 2011, Fuligni, 2019). Other observers have speculated that activation in reward processing regions while giving to others may be a neural correlate of the “warm glow” or positive affect experienced while helping others (Keltner et al., 2014, Moll et al., 2006). The lack of either linear or non-linear age trends in VS and VTA activation is in contrast to studies that have observed peaks in such activation during middle adolescence when witnessing close friends or parents receive rewards (Braams and Crone, 2017b, Schreuders et al., 2021). The lack of 16- to 18-year-old participants may have contributed to the absence of similar non-linear trends in the current study, and additional studies need to determine whether activation in reward processing regions change or remain stable across adolescence when making the decision to give to others at a cost to oneself.

In contrast to the engagement of regions associated with cognitive control and reward processing, there was either no activation (dmPFC) or lower activation (TPJ, pSTS) in those associated with social cognition when adolescents gave money to others as compared to control trials. Although some studies have observed activation in these regions while individuals engage in prosocial behavior, functional analyses showing more activation of the dmPFC, TPJ, and pSTS often include tasks that explicitly require more mentalizing or empathy (Bellucci et al., 2020b, Blakemore and Mills, 2014). More activation also has been observed at the time that adolescents view others receiving rewards due to their correct guessing (Braams et al., 2014a, Braams et al., 2014b). Our task – a dictator game that did not explicitly manipulate recipients’ need or considerations of trust – likely did not include all the social-cognitive demands of other types of prosocial behavior (Keltner et al., 2014). An additional possibility is that the role of networks associated with social cognition is more evident in analyses of individual differences in giving behavior or in connectivity with other regions, as has been found in other studies employing dictator-type giving tasks (Do and Telzer, 2019, Telzer et al., 2011).

The lack of target differences in any neural activation suggests that the brain regions analyzed in this study engage similarly across various potential recipients of prosocial giving during adolescence. Comparable findings were obtained in a recent study by van de Groep et al. (2022) and together with ours, suggest that the developing adolescent brain will respond similarly to the same prosocial decision regardless of whether the recipients are friends, family members, or strangers. It is possible that there would be more differences in activation if additional information about the length, stability, and quality of the relationships with the target caregivers and friends were assessed or even manipulated, as done by Schreuders et al., 2018, Schreuders et al., 2019 in their studies of giving to friends and disliked peers. Another study that observed target differences included young adults more typical of the very top of the age range in our study (van de Groep et al., 2020a). Additional studies that extend from adolescence into and through young adulthood would help to clarify whether differentiation in neural activation according to the recipient becomes more typical during early and later 20 s, a time of continued maturation of key networks (e.g., cognitive control) involved in prosocial behavior. Finally, network and connectivity analyses between regions and systems could shed more light on differentiation in neural activation patterns according to target, such as when giving to strangers and other out-group members (Bellucci et al., 2020a, Do and Telzer, 2019).

Results should be considered in light of the current study’s limitations. Although we employed a well-validated giving task that has successfully been used with adolescents in other studies (Do et al., 2019, Telzer et al., 2014, Telzer et al., 2015), adolescents of different ages may assign different value to the same amounts of money. The simplicity of the task facilitated the inclusion of younger ages and helped to constrain the interpretation of our findings, but our use of the dictator game did not include more complex features of prosocial behavior in everyday life, such as the need of the recipient or the potential for reciprocity. Although the fMRI modeling of decisions to make costly donations to others provided a focus on actual, voluntary decisions to help others, we were limited to including in the analyses those participants who made enough of those decisions to allow for more reliable parameter estimations. Our large sample and wide age-range allowed for better estimation of both linear and non-linear age trends, but the cross-sectional nature of the study limits our conclusions about actual developmental change. Finally, the gap in ages between 16 and 18 years meant that the age differences during this period could not be estimated.

In conclusion, our results provide further evidence for increased differentiation in prosocial behavior across the teenage years by which older adolescents increasingly give more to known others as compared to strangers. This differentiation in behavior was associated with age-related increases in brain activation in the lateral prefrontal cortex, suggesting an important role played by cognitive control networks in the developmental changes in prosocial behavior during the adolescent years. Further research should examine connections between these systems and the consistently active reward-processing regions, as well as potential interactions with regions associated with social cognition, in order to better understand how these neural systems work together to support the increasingly complex social reasoning and behavior that typifies the adolescent period.

## Data Statement

Data will be made publicly available.

## Funding

Support for this research was provided the 10.13039/100000001National Science Foundation Award (#1551952) to A.J.F., N.I.E., and A.G., and the Eunice Kennedy Shriver National Institute of Child Health & Human Development (NICHD) (#1R01HD093823–01) awarded to A.J.F., N.I.E., and A.G.

## Data Availability

Data will be made available on request.
