# Supplementary material for: Giving to others and neural processing during adolescence
Source: Dev Cogn Neurosci. 2022 Jun 22;56:101128. doi: 10.1016/j.dcn.2022.101128 (PMC9249997; doi:10.1016/j.dcn.2022.101128)
Supplement: Supplementary file 1 — Supplementary material [file mmc1.docx]

Supplemental Information: Table of Contents

| **Table S1**…………………………………………………………………………………………...……….**2** |
| --- |
| ROIs Implicated in Cognitive Control: Activation during Giving by Age and Target *before* Bonferroni Correction |
| **Table S2**……………………………………………………………………………………………...…….**3** |
| ROIs Implicated in Cognitive Control: Activation during Giving by Age and Target *before* Bonferroni Correction |
| **Table S3**………………………………………………………………………………………...………….**4** |
| ROIs Implicated in Reward Processing: Activation during Giving by Age and Target *before* Bonferroni Correction |
| **Table S4**……………………………………………………………………………………………………**5** |
| Whole Brain Results Positively Associated with Linear Age Comparing Costly Giving Trials > Control Trials |
| **Giving Task Pre-Scan Instructions**……………………………...……………………………………….**7** |
|  |
|  |
|  |

**Table S1.** *ROIs Implicated in Cognitive Control: Activation during Giving by Age*

|  | dlPFC Mean BOLD Activation  (*n* = 218) | | vlPFC Mean BOLD Activation  (*n* = 208) | |
| --- | --- | --- | --- | --- |
|  | b | SE | b | SE |
| Intercept | 0.31* | 0.04 | -0.03 | 0.04 |
| Age | 0.05*** | 0.01 | 0.02** | 0.01 |
| Age^2^ | -0.01** | 0.002 | -- | -- |
| Caregiver | -0.09 | 0.05 | -0.05 | 0.05 |
| Friend | -0.07 | 0.05 | -0.06 | 0.05 |

*Note*. Age was mean-centered at 13.05 years. **p* < .05, ***p* < .01, ****p* < .001. Quadratic age is denoted as Age^2^.

Results for analyses of ROIs, before Bonferroni correction for family-wise error. Analyses examined linear and non-linear effects of age. Models with higher-order terms are presented only if those higher-order term were statistically significant at *p* < .05.

**Table S2.** *ROIs Implicated in Social Cognition: Activation during Giving by Age and Target*

|  | dmPFC Mean BOLD Activation  (*n* = 208) | | TPJ Mean BOLD Activation  (*n* = 218) | | pSTS Mean BOLD Activation  (*n* = 218) | | |
| --- | --- | --- | --- | --- | --- | --- | --- |
|  | b | SE | b | SE | b | | SE |
| Intercept | 0.09* | 0.04 | -0.19*** | 0.04 | -0.17*** | 0.04 | |
| Age | 0.02 | 0.01 | 0.01 | 0.01 | 0.004 | 0.01 | |
| Caregiver | -0.11 | 0.05 | -0.003 | 0.05 | -0.03 | 0.05 | |
| Friend | -0.08 | 0.05 | -0.004 | 0.05 | -0.04 | 0.05 | |
| Caregiver x Age | -0.01 | 0.02 | -0.03 | 0.02 | 0.001 | 0.02 | |
| Friend x Age | -0.04* | 0.02 | -0.04* | 0.02 | -0.04* | 0.02 | |

*Note*. Age was mean-centered at 13.05 years. **p* < .05, ***p* < .01, ****p* < .001

Results for analyses of ROIs, before Bonferroni correction for family-wise error. Analyses examined linear and non-linear effects of age, as well as interactions of those effects with target (i.e., caregiver). Models with higher-order terms are presented only if those higher-order term were statistically significant at *p* < .05.

**Table S3.** *ROIs Implicated in Reward Processing: Activation during Giving by Age and Target*

|  | VS Mean BOLD Activation  (*n* = 219) | | VTA Mean BOLD Activation  (*n* = 187) | |
| --- | --- | --- | --- | --- |
|  | b | SE | b | SE |
| Intercept | 0.10*** | 0.03 | 0.13** | 0.04 |
| Age | 0.02* | 0.01 | -0.01 | 0.01 |
| Caregiver | -0.05 | 0.03 | 0.05 | 0.06 |
| Friend | -0.01 | 0.03 | 0.06 | 0.06 |
| Caregiver x Age | -0.001 | 0.01 | 0.03* | 0.02 |
| Friend x Age | -0.02* | 0.01 | 0.02 | 0.02 |
| Age^2^ | -- | -- | 0.003 | 0.002 |
| Caregiver x Age^2^ | -- | -- | -0.01* | 0.003 |
| Friend x Age^2^ | -- | -- | -0.01* | 0.003 |

*Note*. Age was mean-centered at 13.05 years. **p* < .05, ***p* < .01, ****p* < .001. Quadratic age is denoted as Age^2^.

Results for analyses of ROIs, before Bonferroni correction for family-wise error. Analyses examined linear and non-linear effects of age, as well as interactions of those effects with target (i.e., caregiver). Models with higher-order terms are presented only if those higher-order term were statistically significant at *p* < .05.

**Table S4.** *Whole Brain Activation Positively Associated with Linear Age*

| **Area of Activation** | **MNI Coordinates** | | | | **Test Statistic** | **Cluster Size** |
| --- | --- | --- | --- | --- | --- | --- |
| **Costly Giving > Control Trials** | **x** | **y** | **z** | ***t*** | |  |
| R Inferior Parietal Lobule | 36 | -55 | 50 | 7.83 | | 863 |
| L Angular Gyrus | -30 | -55 | 41 | 6.29 | | 542 |
| R Inferior Frontal Gyrus | 48 | 11 | 26 | 6.51 | | 454 |
| R Inferior Frontal Gyrus (p. Triangularis) (i.e., dlPFC) | 42 | 35 | 17 | 3.93 | | 454 |
| R Fusiform Gyrus | 36 | -79 | -13 | 5.83 | | 448 |
| L Inferior Frontal Gyrus (p. Triangularis) (i.e., dlPFC) | -48 | 8 | 29 | 5.84 | | 329 |
| L Fusiform Gyrus | -42 | -76 | -10 | 5.42 | | 320 |
| R Middle Frontal Gyrus | 36 | 5 | 59 | 6.35 | | 134 |
| L Superior Medial Gyrus | -6 | 32 | 44 | 4.67 | | 133 |
| R Inferior Frontal Gyrus (p. Orbitalis) | 33 | 23 | -1 | 5.07 | | 67 |

*Note: Names are from the AAL toolbox in SPM. See <https://identifiers.org/neurovault.collection:12218> to examine the unthresholded activation maps.

MNI coordinates of local maxima that were active for the t-test for Costly Giving Trials > Control Trials. Results are calculated using a voxel-wise height threshold of *p* < .001 (uncorrected) combined with a cluster-level extent threshold of *p* < .05, corrected for multiple comparisons using the family-wise error (FWE) rate. Contrasts examining whole brain activation by giving target (i.e., caregiver) did not survive cluster correction. The strongest area of activation is listed for each cluster as well as any areas that overlapped with the a priori regions of interest (i.e., dlPFC).

**Giving Task Pre-Scan Instructions:**

**Choosing a Friend:** “*While you're here, we'll be asking you some questions about a few different people in your life. For example, we'd like you to pick a close friend. This should NOT be a boyfriend, girlfriend, or someone you are related to, but someone you feel really close to and consider a good friend of yours. Can we have the first name, gender and age of this friend?*”

**Choosing a Caregiver:** “*Great. We'd also like you to pick a primary caregiver. This person should be an adult who generally takes/took care of you and had a big part in raising you. You may have had more than one person fill this role for you while growing up, but we'd like you pick just one of these people. This could be your mother, your father, or someone along those lines. This person should also be someone you're still in touch with. Who would you like to pick?”*

*“During the tasks today, you’ll be playing to win money for your friend and caregiver. At the end of today’ visit, we’ll tally up your winnings from the games you played, and put the money you earned for each person in these envelopes for you to give them.”*

**Stranger Information:** “*You will also be playing to win money for a future participant, but because we haven’t met them yet and don’t know their name, we are just going to call them Alex. Do you have any close friends or family members named Alex?*”

If the participant has a familiar friend or family member with that name:

“*OK. How about Taylor?*”

“*This obviously isn’t their real name, but you will be playing for a real participant that will be coming in sometime after your session today. Another past participant has already played for you just like you’re playing for someone else today. Whatever they earned for you during their session will be included in your final payment. Similarly, whatever you earn for the next participant will be given to them as part of their final payment. Does that make sense?*”
